# Regulatory Mechanism of lncRNAs in M1/M2 Macrophages Polarization in the Diseases of Different Etiology

**DOI:** 10.3389/fimmu.2022.835932

**Published:** 2022-01-25

**Authors:** Ping Jiang, Xiaopeng Li

**Affiliations:** ^1^ Guanghua Clinical Medical College, Shanghai University of Traditional Chinese Medicine, Shanghai, China; ^2^ Department of Rheumatology, Shanghai Guanghua Hospital of Integrated Traditional Chinese and Western Medicine, Shanghai University of Traditional Chinese Medicine, Shanghai, China; ^3^ Department of Neurology, Rizhao Hospital of Traditional Chinese Medicine, Rizhao, China; ^4^ Integrated Traditional Chinese and Western Medicine, Shandong University of Traditional Chinese Medicine, Jinan, China

**Keywords:** long noncoding RNAs, regulation, macrophages, polarization, diseases

## Abstract

Precise expression and regulation of genes in the immune system is important for organisms to produce strong immunity towards pathogens and limit autoimmunity. In recent years, an increasing number of studies has shown that long noncoding RNAs (lncRNAs) are closely related to immune function and can participate in regulating immune responses by regulating immune cell differentiation, development, and function. As immune cells, the polarization response of macrophages (*Mφs*) plays an important role in immune function and inflammation. LncRNAs can regulate the phenotypic polarization of *Mφs* to M1 or M2 through various mechanisms; promote pro-inflammatory or anti-inflammatory effects; and participate in the pathogenesis of cancers, inflammatory diseases, infections, metabolic diseases, and autoimmune diseases. In addition, it is important to explore the regulatory mechanisms of lncRNAs on the dynamic transition between different *Mφs* phenotypes. Thus, the regulatory role of lncRNAs in the polarization of *Mφs* and their mechanism are discussed in this review.

## Introduction

**Figure 1:**
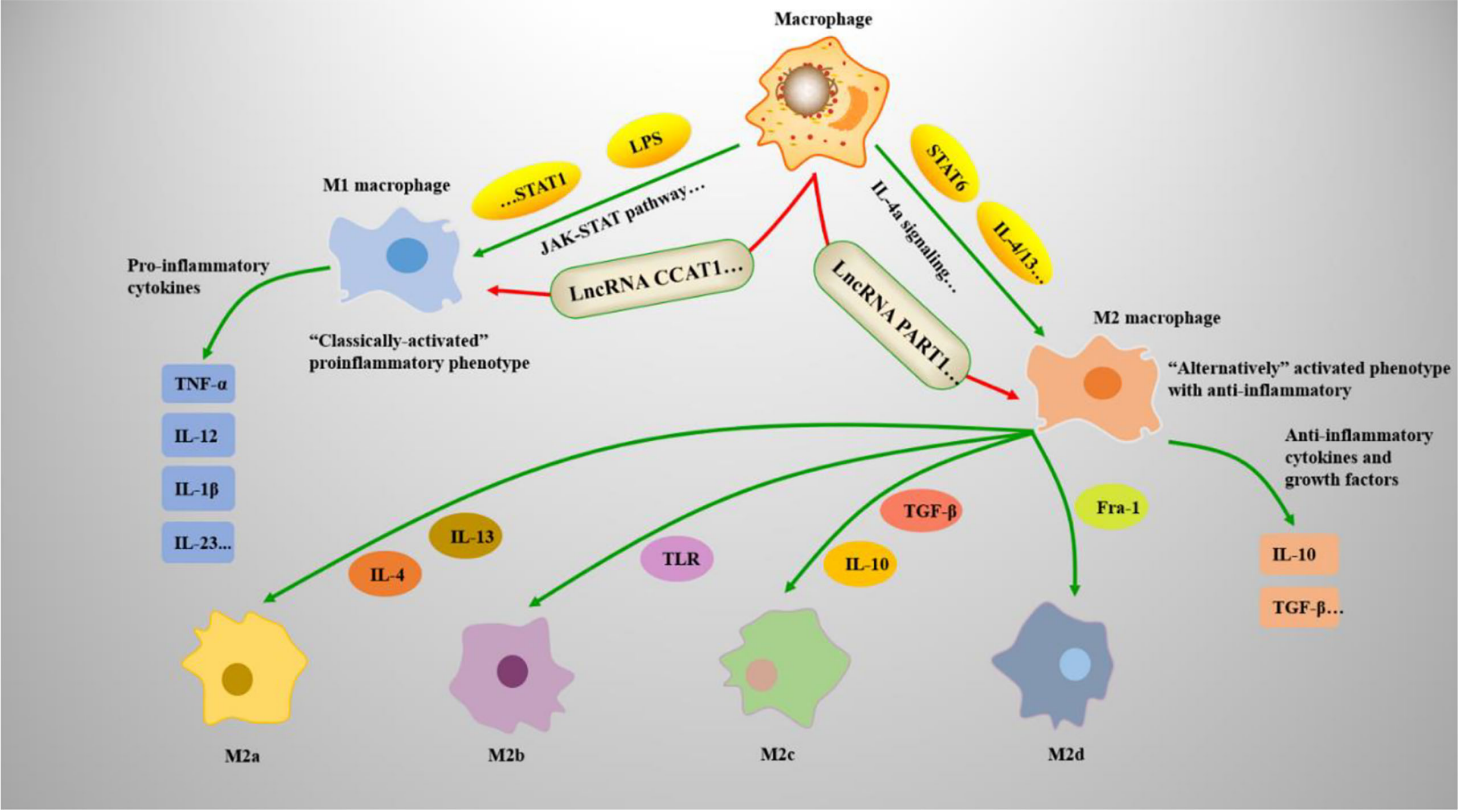
*Mφs* polarization mechanisms and types. *Mφs* adopt a proinflammatory M1 phenotype or anti-inflammatory M2 phenotype. (1) Under the action of the JAK-STAT pathway, STAT1 dimerizes and reacts with interferon (IFN)-γ to induce M1-associated genes, such as TNF-α and IL-12. Lipopolysaccharide (LPS) also induces M1-associated genes by forming the IFN-stimulated gene factor 3 (ISGF3) complex. (2) IL-4, IL-13, and STAT6 are associated with M2 *Mφ* polarization. STAT6 mediates IL-4a signaling and regulates M2-associated genes, such as IL-10 and TGF-β; (3) the M2 phenotype can differentiate into M2a, M2b, M2c, and M2d under the induction of related genes.

Macrophages (*Mφs*) are vital antigen-presenting cells with high heterogeneity and plasticity in the human immune system (1). *Mφs* can induce multiple polarization processes and exert different functions depending on the conditions (2). The local cytokine environment can lead to polarization of *Mφs*. Therefore, according to their expression of cell surface markers, the production of specific factors, and biological activities, *Mφs* are classified into the M1-like and M2-like types (3). M1 and M2 polarization is a remarkable characteristic of *Mφs* involved in numerous biological processes. M1 *Mφs are* known as classical *Mφs* and are activated by bacterial lipopolysaccharide or interferon (IFN)-γ. To produce and secrete higher levels of proinflammatory cytokines, including tumor necrosis factor (TNF)-α, interleukin (IL)-6, IL-12, and cyclooxygenase-21. M1 Mφs have robust antimicrobial functions and inhibit tumor growth (4). In M2 *Mφs*, signal transducer and activator of transcription 6 (STAT6) is activated by IL-4 receptor α (IL-4Rα) and M2 *Mφs are* polarized by Th2 cytokine IL-4/IL-13. M2 *Mφs* have anti-inflammatory and strong phagocytic abilities to eliminate apoptotic cells and are useful for chronic infection treatment and wound healing (5). Moreover, according to the different stimuli received, M2 *Mφs* can be further divided into four different types: M2a, M2b, M2c, and M2d (6) (See **Figure 1**).

Precise regulation of *Mφs* polarization is a key step in controlling immune system function, clearing pathogens, and defending against external evil. The evidence accumulated in the past decade shows that lncRNAs are widely expressed and plays a key role in gene regulation. LncRNAs are a group of non-coding RNAs >200 nucleotides in length and composed of molecules transcribed by different types of RNA polymerase II (7). LncRNAs could regulate chromatin function, change the stability and translation of cytoplasmic mRNAs, and interfere with the signaling pathways. These functions influence gene expression in different biological and pathological environments, such as immunoreactive diseases (8). In addition, lncRNAs are responsible for the integrity of nuclear structure and can regulate the expression of nearby genes or genes in other parts of the cell by interacting with proteins, RNAs and DNAs (7). LncRNAs are a potential biomarker (9) and act as crucial regulators of *Mφs* polarization in response to intracellular or extracellular stimulation. various lncRNAs exhibit different functions in *Mφs* infiltration and polarization and regulate the secretion of inflammatory cytokines released by *Mφs* (10). Recent studies indicated that lncRNAs could participate in M1 or M2 polarization and exert multifunctional roles in disease development (11) (See **Tables 1**, **2**).

**Table 1:** LncRNAs regulate polarization types of *Mφs* and targets in cancers.

LncRNAs	Polarization Types	Targets	Diseases	References
LncRNA ANCR	M1	FOXO1	Gastric cancer	(12)
LncRNA CCAT1	M1	MiR-148A	Colon cancer	(13).
LncRNA GAS5	M1	Unknown	Colorectal cancer	(14)
LncRNA LNMAT1	M1	CCL2	Bladder cancer	(15)
LncRNA p21	M1	MDM2/p53/NF-κB/STAT3	Breast cancer	(16)
LncRNA GNAS-AS1	M2	MiR-433-3p	Breast cancer	(17)
LncRNA BCRT1	M2	MiR-1303/PTBP3	Breast cancer	(18)
LncRNA SNHG1	M2	STAT6	Breast cancer	(19)
LncRNA 00337	M2	CD163/ARG1	Breast cancer	(20)
LncRNA 00514	M2	Jagged1/Notch	Breast cancer	(21)
LncRNA GAS5	M2	PTEN	Hepatocellular carcinoma	(22)
LncRNA PART1	M2	MiR-372-3p/TLR4	Hepatocellular carcinoma	(23)
LncRNA DLX6-AS1	M2	MiR-15a-5p/CXCL17	Hepatocellular carcinoma	(24)
LncRNA TP73-AS1	M2	MiR-539/MMP-8/TGF-β1	Hepatocellular carcinoma	(25)
LncRNA CRNDE	M2	CD163	Liver cancer	(26)
LncRNA HLA-F-AS1	M2	MiR-375/PFN1	Colorectal cancer	(27)
LncRNA NBR2	M2	Arg-1/CD163/CD206	Colorectal cancer	(28)
LncRNA PTTG3P	M2	HIF1A	Colorectal cancer	(29)
LncRNA RPPH1	M2	TUBB3	Colorectal cancer	(30)
LncRNA MIR155HG	M2	MiR-650/ANXA2	Colorectal cancer	(31)
LncRNA RP11-361F15.2	M2	MiR-30c-5p/CPEB4	Osteosarcoma	(32)
LncRNA LOC100129620	M2	MiR-335-3p/CDK6	Osteosarcoma	(33)
LncRNA NEAT1	M2	MiR-214/B7-H3	Endometrial cancer	(34, 35)
LncRNA NIFK-AS1	M2	MiR-146a	Endometrial cancer	(36)
LncRNA-MM2P	M2	STAT6	Tumorigenesis	(37)
LncRNA CASC2c	M2	FX	Brain tumor	(38)
LncRNA LINC01140	M2	MiR-140-5p/FGF9	Bladder cancer	(39)
LncRNA GNAS-AS1	M2	MiR-4319/NECAB3	Non-small cell lung cancer	(40)
LncRNA XIST	M2	TCF-4	Lung cancer	(41)
LncRNA DCST1-AS1	M2	NF-κB	Oral Squamous Cell Carcinoma	(42)
LncRNA FGD5-AS1	M2	MiR-129-5p/BST2	Cervical cancer	(43)
LncRNA C00467	M2	MiR-494-3p/STAT3	Prostate cancer	(44)
LncRNA HCG18	M2	MiR-875-3p/KLF4	Gastric cancer	(45)
LncRNA TUC339	M1/M2	CXCR	Hepatocellular carcinoma	(46)
LncRNA COX-2	M1/M2	IL-12/iNOS/TNF-α/IL-10/Arg-1/Fizz-1	Hepatocellular carcinoma	(47)
LncRNA Ma301	M1/M2	Caprin-1/Akt/Erk1	Hepatocellular carcinoma	(48)
LncRNA LBX1-AS1	Macrophage	MiR-182-5p/FOXO3	Oral squamous cell carcinoma	(49)
LncRNA Xist	M1/M2	MiR-101-3p/KLF6/C/EBPα	Breast cancer	(50)
LncRNA Xist	M1/M2	MiR-101-3p/KLF6/C/EBPα	Ovarian tumor	(50)

**Table 2:** LncRNAs regulate polarization types of *Mφs* and targets in other diseases.

LncRNAs	Polarization Types	Targets	Diseases	References
LncRNA GAS5	M1	STAT1	Diabetic wound healing	(51)
LncRNA HCG18	M1	MiR−146a/TRAF6	Diabetic peripheral neuropathy	(52)
LncRNA GAS5	M1	MiR‐455‐5p/SOCS3	Childhood pneumonia	(53)
LincRNA p21	M1	NF-κB/p65	Acute respiratory distress syndrome	(54)
LncRNA AFAP1-AS1	M1	MiR-214	Aortic valve calcification	(55)
LncRNA NRON	M1	IL-12	Atrial fibrillation	(56)
LncRNA GBP9	M1	MiR-34a/SOCS3	Spinal cord injury	(57)
LncRNA XIST	M1	MiR‐376c‐5p/OPN	Osteoarthritis	(58)
LncRNA MEG8	M1	MiR-181a-5p/SHP2	Henoch-Schonlein purpura	(59)
LncRNA H19	M1	CCL-2/CCR-2	Cholestatic liver diseases	(60)
LncRNA H19	M1	KDM6A	Rheumatoid arthritis	(61)
LncRNA PVT1	M1	MiR-29a/HMGB1	Sepsis	(62)
LncRNA PTPRE-AS1	M2	PTPRE	Pulmonary allergic inflammation	(63)
LncRNA NKILA	M2	NF-kB	Asthma	(64)
LncRNA BAZ2B	M2	IRF4	Asthma	(65)
LncRNA AK085865	M2	Unknown	Allergic asthma	(66)
LncRNA AK085865	M2	MiR-192	Coxsackievirus B3 -induced viral myocarditis	(67)
LncRNA MEG3	M2	MiR-223/TRAF6	Viral myocarditis	(68)
LncRNA NRON	M2	MiR-23a	Atrial fibrillation	(56)
LncRNA SNHG20	M2	STAT6	Nonalcoholic fatty liver disease	(69)
LncRNA NEAT1	M2	MiR-125a-5p/TRAF6/TAK1	Acute kidney injury	(70)
LncRNA ASLNCS5088	M2	GW4869	Hypertrophic scar	(71)
LncRNA NEAT1	M2	MiR-224-5p/IL-33	Spinal cord injury	(72)
LncRNA TUG1	M2	MiR-9-5p/SIRT1	Sepsis	(73)
LncRNA NEAT1	M2	MiRNA-148a-3p	Age-related macular degeneration	(74)
LncRNA Gomafu	M2	Unknown	Obesity-induced chronic inflammation	(75)
LncRNA Gas5	M2	TRF4	Multiple sclerosis	(76)
LncRNA MIR155HG	M1/M2	MiR-155	Chronic obstructive pulmonary disease	(77)
LncRNA Gm16410	M1/M2	PI3K/AKT	Lung inflammation	(78)
LncRNA KCNQ1OT1	M1/M2	MiR-21a-5p	Osteoarthritis	(79)
LncRNA DYNLRB2-2	Macrophage	ABCA1/SIRT3	Atherosclerosis	(80)
LncRNA MAARS	Macrophage	HuR	Atherosclerosis	(81)
LncRNA RAPIA	Macrophage	MiR-183-5p/ITGB1	Atherosclerosis,	(82)
LncRNA NEAT1	Macrophage	MiR‐342‐3p	Atherosclerosis	(83)
LncRNA UCA1	Macrophage	MiR‐206	Atherosclerosis	(84)
LncRNA MIAT	Macrophage	NLRP3	Cardiovascular disease	(85)
LncRNA Dnm3os	Macrophage	H3K9ac	Diabetes Mellitus	(86)
LncRNA Lethe	Macrophage	NOX2	Diabetic wound healing	(87)
LncRNA FTX	Macrophage	MiR‐545/Tim‐3	HBV‐related cirrhosis	(88)
LncRNA H19	Macrophage	Rho-GTPase CDC42/RhoA	Biliary atresia	(60)
LncRNA Maclpil	Macrophage	LCP1	Ischemic stroke	(89)
LncRNA IGHCγ1	Macrophage	MiR-6891-3p/TLR4	Osteoarthritis	(90)
LncRNA Mist	Macrophage	PARP1	Obesity	(91)
LncRNA SAF	Macrophage	Caspase-3/7	HIV-1 infection	(92)
LncRNA GAPLINC	Macrophage	NF-κB	Sepsis	(93)
LncRNA Gm16410	Macrophage	SRC/PI3K/AKT	Lung inflammation	(78)
LncRNA CRNDE	Macrophage	NLRP3c	IgA Nephropathy	(94)

Here, we review the current understanding of lncRNA functions in *Mφs* polarization. Using different disease models, we reviewed that different lncRNAs regulate the polarization of *Mφs via* different signaling pathways and act on different targets; we also explain the mechanism of these effects.

## LncRNAs Regulate *Mφs* Polarization in Various Cancers

**Figure 2:**
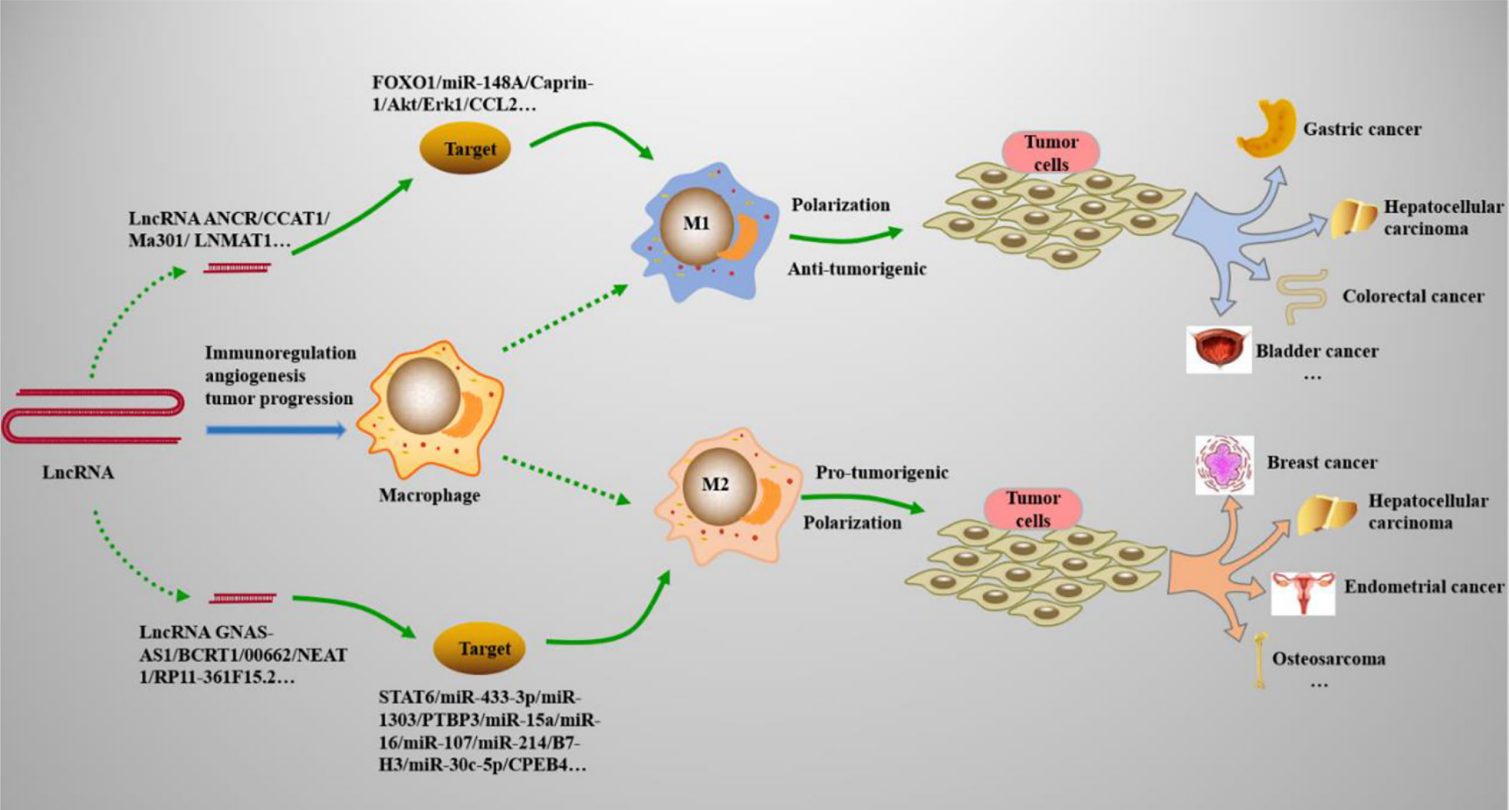
LncRNAs regulate *Mφs* polarization in cancers. Many types of lncRNAs play important roles in cancers by acting on the M1 and M2 polarization processes of *Mφs*. (1) M1 polarization can resist tumor cells and play an immunomodulatory role in tumor microenvironments, such as gastric cancer, bladder cancer, and colorectal cancer. (2) M2 polarization promotes the growth, migration, and proliferation of tumor cells, and is involved in the progression of cancers, such as breast cancer, hepatocellular carcinoma, and osteosarcoma.

Tumor-associated macrophages (TAMs), which are specialized phagocytic cells, play an important role in the pathology and pathogenesis of cancers and are responsible for modulating the tumor microenvironment (TME) (95) (See **Table 1**). The microenvironment signals exposed to *Mφs* are the key factors that determine the phenotype of *Mφs*; these microenvironment signals selectively regulate their functions, including M1 and M2 polarization (96). At present, TAMs are mainly divided into two subtypes (See **Figure 2**): M1 TAMs of classical activation pathway and M2 TAMs of alternative activation pathway. M1 TAMs have significant anti-tumor effect. In the TME, it can identify tumor cells and kill tumor cells through the corresponding signaling pathway. According to the different pathways of action, it can be divided into direct killing mechanism and antibody-dependent cytotoxicity (ADCC) mechanism (97). M2 TAMs can significantly promote tumor growth. First of all, M2 TAMs secrete cytokines such as IL-6 and CXCL-8 into the TME and directly promotes the growth of tumor cells (98). Secondly, M2 TAMs can also block the inducible nitric oxide synthase (iNOS) pathway, reduce the synthesis of NO, accelerate the production of polyamines, and then promote the proliferation of tumor cells (99). M2 TAMs also promote angiogenesis of tumor cells by secreting vascular endothelial growth factor (VEGF) and IL-17, and can release matrix metalloproteinase (MMP)-2, MMP-9 and other substances to degrade extracellular matrix, promote vascular endothelial migration and induce angiogenesis (100, 101). M1 and M2 TAMs existed in all stages of the tumor, mainly M1 in the early stage and M2 in the middle and late stages of the tumor. With the progression of tumor, M1 is gradually polarized to M2, and the increase number of M2 TAMs also indicates a poor prognosis. Clinical studies have confirmed that M1 TAMs polarize to M2 TAMs induced by proliferator-activated receptor (PPAR)-γ. The increase in the proportion of M2 TAMs are closely related to tumor cell proliferation and tumor tissue vascular density (MVD) (102). A new study has shown that the inhibition of nuclear factor-kappa B (NF-κB) activity and the increase of STAT3 activity in *Mφs* represent a tumor-mediated mechanism that triggers M1/M2 deviation (103). Moreover, lncRNAs are increasingly appreciated as important regulators of gene expression (104), and multiple studies have showed (105, 106) that lncRNAs have a significant role in the regulation of TAM polarization in TME and functions principally as diagnostic and therapeutic biomarkers. Because lncRNAs are highly stable, specific and easy to detect, and can be expressed abnormally under different pathophysiological conditions (107); it can be used as a regulatory molecule of gene expression, acting as oncogenes or tumor suppressors, thus playing a role and directly involved in the occurrence of cancer, apoptosis, cell migration and invasion (108, 109). As a potential biomarker for diagnosis and prediction, lncRNAs have a wide range of functions in the occurrence and development of various human diseases, especially in the occurrence and development of many cancers.

### Respiratory System Cancers

Lung cancer is the most common respiratory cancer, with non-small cell lung cancer (NSCLC) accountings for approximately 85% of all lung cancers as the most common cancer worldwide. Increasing evidence has shown that *Mφs* and lncRNAs are involved in the development of inflammation and malignant transformation of tumor cells. LncRNAs are also involved in the pathogenesis of lung cancer. Li et al. (40) used lncRNA*GNAS-AS1*, to explore the role of *GNAS-AS1* in the development of NSCLC. These experimental studies showed that lncRNA*GNAS-AS1* was highly expressed in M2 *Mφs* and NSCLC cell lines, which was significantly higher than that in M1 *Mφs*, and promoted the polarization of M2 *Mφs* and progression of NSCLC by directly inhibiting miR4319 and increasing the level of N-terminalEF-handcalciumbindingprotein3 (NECAB3). In addition, lncRNA*GNAS-AS1* was also highly expressed in lung TAMs and clinical tumor tissues. Therefore, lncRNA*GNAS-AS1* promoted the polarization of M2 *Mφs* and induced the progression of NSCLC through the miR-4319/NECAB3M2 axis in the tumor microenvironment, showing potential as a target for treating NSCLC. Another study on lung cancer confirmed that lncRNA *XIST* can also play a role by regulating *Mφs*. The expression of lncRNA *XIST* in M2 *Mφs* was significantly higher than that in M1 *Mφs*. Down-regulation of lncRNA *XIST* could significantly inhibit IL-4-induced M2 polarization, resulting in downregulation of M2 specific markers such as IL-10 and CD163. Due to the direct interaction between T-cell-specific transcription factor (TCF)-4 and lncRNA *XIST*, this inhibition was stopped by the overexpression of TCF-4. However, lncRNA XIST knockout can restore IL-4-induced M2 polarization, further indicating the importance of TCF-4 in M2 *Mφs* and TAMs-induced proliferation, migration and invasion of lung cancer cells. The results showed that lncRNA *XIST* positively regulated the M2 polarization and interacted with TCF-4, thus regulating the development of lung cancer (41).

### Urinary System Cancers

Prostate cancer (PC) is one of the most common tumors in the urinary system, and methods for its diagnosis and treatment have rapidly progressed in recent years; however, therapeutic effects remain unsatisfactory (110). Recently, the therapeutic value of lncRNAs in regulating *Mφs* in the TME has been widely explored, and a Chinese research team has found (44) that lncRNA *LINC00467* may be beneficial for treating PC. Additionally, the lncRNA *LINC00467* mainly exists in the cytoplasm, and its expression is upregulated in PC tissues and cells to enhance polarization of the M2 phenotype *via* the miR-494-3p/STAT3 axis. While M2 polarization can activate the STAT3 pathway, enhance the tumor enhancement of M2, and thus promote the growth and migration of PC cells (44).

Compared with PC, bladder cancer is among the most serious malignant tumors worldwide. Although the incidence of muscle-invasive bladder cancer (MIBC) is low, the overall mortality rate is high. Chen et al. (15) found that a new lncRNA named as *LNMAT1* through sequencing of five MIBC organizations. Its expression in MIBC tissues was significantly upregulated and positively correlated with the intensity of *Mφs* infiltration around the tumors. Further experiments showed that overexpression of *LNMAT1 in vivo* activated *Mφs* polarization by mediating heterogeneous nuclear Ribonucleoprotein L (hnRNPL) to promote the transcription of its target gene, CCL2. Furthermore, this lncRNA can induce the expression of CCL2 in bladder cancer cells, upregulate CCL2 to stimulate TAMs to secrete VEGF-C, participate in lymphogenesis, promote lymphatic metastasis and lymphangiogenesis, and accelerate the invasiveness of bladder cancer cells. These findings elaborate that *LNMAT1* regulates the polarization of TAMs and mediates lymphatic metastasis, revealing a carcinogenic role for *LNMAT1* in bladder cancer progression. Differential genes of lncRNA *LINC01140* and fibroblast growth factor 9 (FGF9) were found in another study of an online microarray expression profile (GSE77952) of MIBC (39). By the analysis of polymerase chain reaction (PCR) and enzyme linked immunosorbent assay (ELISA), revealed a positive correlation between the expression of *LINC01140* and FGF9. FGF9 and *LINC01140* were highly expressed in M2 *Mφs*; after knocking down FGF9 and *LINC01140*, the viability and invasive ability of MIBC cells were significantly inhibited, leading to a decrease in the M2 markers IL-10 and Arg1 and weakened M2 polarization. In addition, by using the competing endogenous RNA mechanism, it was found that inhibition of miR-140-5p significantly reversed the expression and effects of *LINC01140* and FGF9, thus affecting M2 polarization. Finally, *LINC01140*, miR-140-5p and FGF9 form a lncRNA-miRNA-mRNA mechanism axis, which influences M2 polarization by regulating the TME, thereby affecting the progression of MIBC.

### Digestive System Cancers

Oral squamous cell carcinoma (OSCC) is a digestive system cancer that is also a common type of head and neck cancer. The occurrence and progress of OSCC are closely related to *Mφs*. M2 *Mφs* accelerate the progression of OSCC through a complex cross-talk mechanism and are regulated by lncRNAs. The NF-κB signaling plays an important role in tumor development. Ai et al. (42) found that deletion of lncRNA *DCST1AS1* hindered the progression of OSCC by inhibiting the NF-κB signaling pathway. Its overexpression can cause M2 polarization to can promote the growth and differentiation of OSCC cells and accelerate the development of cancer by regulating the tumor immune environment. Another lncRNA, *LBX1-AS1*, is secreted by RBPJ-overexpression (RBPJ-OE) *Mφs* and belongs to the exocrine RNA. Its mechanism is mediated *via* the miR-182-5p/FOXO3 pathway. As a competing endogenous RNA, *LBX1-AS1* stimulates *Mφs* by acting on miR-182-5p and fork head box protein O3 (FOXO3), causing *Mφs* polarization and activating *Mφs* to regulate the development of tumor cells (49).

Gastric cancer (GC) is a common disease affecting the digestive system. In recent years, research on GC has mainly focused on the molecular mechanism and attempts to identify new therapeutic targets. LncRNAs and *Mφs* polarization were shown to be closely related to the development of GC. M1 polarization can kill tumor cells and inhibit tumor lymphangiogenesis and angiogenesis (111). Overexpression of lncRNA *ANCR* in *Mφs* significantly reduced the expression of the M1 polarization marker IL-1β and IL-6, thus inhibiting M1 polarization. Co-culture of *Mφs* with GC cells showing high expression of lncRNA *ANCR* resulted in significantly enhanced invasiveness and migration of GC cells. FOXO1 is the target gene of the lncRNA *ANCR*. On one hand, FOXO1 can promote *Mφs* to produce inflammatory factors and induce M1 polarization. In contrast, lncRNA *ANCR* accelerates the ubiquitination of FOXO1 and inhibits its expression. After downregulation of FOXO1, M1 polarization was also inhibited. Based on these results, overexpression of lncRNA *ANCR* can downregulate FOXO1, thus inhibiting M1 polarization and promoting the development of GC cells (12). Other lncRNA can affect GC progression by influencing M2 polarization. M2 *Mφs* can induce GC cells growth, and lncRNA *HCG18* can positively regulate M2 polarization. Specifically, lncRNA *HCG18* can bind to miR-875-3p and target krüppel-like factor4 (KLF4) regulation, as well as upregulate KLF4 by reducing the expression of miR-875-3p in *Mφs*, thereby promoting M2 polarization. M2 *Mφs* have anti-inflammatory effects, secrete inflammatory cytokines to kill tumor cells, slow GC development, and play a role in immune surveillance (45).

Hepatocellular carcinoma (HCC) is a common tumor of the digestive system with a high incidence in Asia. M2 *Mφs* can promote tumor angiogenesis and induce cancer cell metastasis, but the mechanism of *Mφs* polarization is unclear. Four studies have explained the role of this mechanism in HCC development through the regulation of different lncRNAs on M2 polarization. It has been found (26) that M2 *Mφs* can promote human umbilical vein endothelial cell angiogenesis when M2-THP-1 cells are co-cultured with these cells. In mouse model of H22 tumor cells, lncRNA *CENDE* downregulated the expression of CD163, STAT-6, and VEGF by promoting M2 polarization, thus indirectly regulating tumor angiogenesis. In a study of SMCC-7721 cells, the supernatant of TAMs significantly promoted cell invasiveness. The expression of lncRNA *GAS5* is downregulated in M2 *Mφs* and TAMs, which has been shown to be related to *Mφs* polarization in the progression of HCC. This lncRNA plays a regulatory role by regulating the expression of the target protein phosphatase and tensin homolog (PTEN) to inhibit M2 polarization of TAMs in SMCC-7721 cells (22). M2 polarization can also interact with the miR-372-3p/TLR4 axis, thus affecting the delivery of lncRNA *PART1* into liver cancer tissues and cells *via* tumor-derived extracellular vesicles, inducing the upregulation of *PART1* and Toll-like receptor 4 (TLR4) and downregulation of miR-372-3p, and promoting the proliferation and invasion of liver cancer cells. Additionally, miR-372-3p can bind to *PART1* and negatively regulate TLR4, act on extracellular vesicles, and induce M2 polarization in HCC tissues, thus promoting the occurrence of HCC (23). The microRNA-15a-5p/CXCL17 axis is another regulatory pathway of M2 polarization in HCC. A previous study showed (24) that lncRNA *DLX6-AS1*, miR-15a-5p, and CXCL17 were highly expressed in HCC cells, and exosomes were isolated from HCC cells and co-cultured with M2 *Mφs*. The results showed that extracellular ubiquitin induced and intracellular HCC-exosomes transported *DLX6-AS1* to *Mφs*, targeted binding to miR-15a-5p, and regulated CXCL17 expression to stimulate M2 polarization. In addition, Wnt ligands in tumor cells can stimulate M2 polarization, which can jointly accelerate the development and metastasis of HCC cells *in vivo* and *in vitro*. Furthermore, numerous studies have shown that different lncRNAs (lncRNA *COX-2*, lncRNA *TUC339*, and lncRNA *Ma301*) induce M1 and M2 polarization in the tumor microenvironment through different action targets (E-cadherin, caprin-1) and pathways (Akt/Erk1pathway), decrease the levels of iNOS and TNF-α in M1 *Mφs*, and increase the levels of Arg-1 and Fizz-1 in M2 *Mφs*. LncRNAs also promotes the production of various inflammatory factors, chemokines and exocrine, thus acting on the immune response of tumors and inhibiting pathological processes such as immune escape and invasion of HCC. These studies provides a new theoretical basis for treating HCC (46–48).

With the aging of the population and poor lifestyle, the global morbidity and mortality of colorectal cancer (CRC) are increasing each year. In epigenetic studies, lncRNAs were confirmed to be closely related to the pathogenesis of CRC. For example, lncRNA *RP4* functions in CRC by binding to miR7-5p (112). Previous studies also showed that *Mφs* are involved in the pathogenesis of numerous cancers. Is there a regulatory relationship between lncRNAs and *Mφs* that interfere with the progression of CRC? Related studies found that lncRNAs *HLA-F-AS1*, *NBR2*, *PTTG3P*, and *RPPH1* induce the polarization response of tumor-associated *Mφs* by acting on the corresponding targets or pathways, thus participating in the pathological development of CRC. Mechanistically, lncRNA *MIR155HG* (31) and lncRNA *HLA-F-AS1* (27) can competitively bind with miR-650 and miR-375, reverse-regulate the expression of miR-650 and miR-375, and indirectly target annexin A2 (ANXA2) and profilin 1 (PFN1). Inhibiting of miR-650 and miR-375 promoted the expression of ANXA2 and PFN1 in CRC-derived extracellular vesicles. This mechanism further stimulated increased M2 *Mφs* marker expression, induced M2 *Mφs* phenotypic polarization, and accelerated CRC cells invasion. In contrast, lncRNA *NBR2* can activate M1 polarization, inhibit M2 polarization, and regulate the polarization of TAMs to promote the release of inflammatory factors such as TNF-α, IL1-β, and IL-10, thus acting on the TME. However, after knockout of the lncRNA *NBR2* gene, these effects were reversed (28). A similar process occurs with lncRNA *PTTG3P* (29). Under the action of hypoxia-inducible factor-1-α (HIF-1α), the hypoxia environment induces the overexpression of *PTTG3P* to promotes M2 polarization and stimulates tumor development. In addition, exosomes can mediate *Mφs* polarization. Liang et al. (30) found that the expression of lncRNA *RPPH1* is upregulated in CRC, which is related to poor prognosis. In terms of the mechanism, *RPPH1* binds to β-III tubulin and prevents its ubiquitination, which induces the transformation of epithelial-mesenchymal transition and accelerates the metastasis of cancer cells. Further studies showed that *RPPH1* is closely related to exosomes, which can transfer into *Mφs* by exosomes to mediate M2 polarization and promote tumor growth and migration.

### Reproductive System Cancers

Breast cancer occurs following uncontrolled proliferation of breast epithelial cells *via* the action of various carcinogenic factors and is considered as a reproductive system cancer. Modern studies showed that the lncRNA-miRNA-mRNA axis plays an important role in cancer progression. The lncRNA *BCRT1*/miR-1303/PTBP3 and lncRNA *Xist*/miR-101-3p/KLF6/C/EBPα can regulate the progression of breast cancer. In the lncRNA *BCRT1*/miR-1303/PTBP3 axis (18), polypyrimidine tract binding protein 3 (PTBP3) promotes breast cancer, and the expression of lncRNA *BCRT1* in breast cancer tissues is significantly increased, which is related to the poor prognosis of patients. In the mechanism of action, lncRNA *BCRT1* can bind to miR-1303 and act on PTBP3 to inhibit its expression. After overexpression, lncRNA *BCRT1* significantly promotes M2 polarization mediated by exosomes. After M2 polarization, inflammatory factors such as TNF-α and IL-6 are produced, which promote cancer in tumor microenvironment; this effect is more obvious in hypoxic environments. Phenotypic transformation of TAMs mediates tumor immune escape. Zhao et al. (50) found that the expression of lncRNA *Xist* and C/EBPα was upregulated in M1 *Mφs*, whereas the expression of miR-101-3p was downregulated. LncRNA *Xist* knockout inhibited the expression of C/EBPα and KLF6 and induced the transformation from M1 to M2, thus promoting the proliferation and migration of breast cancer cells. Therefore, lncRNA *Xist* competes with miR-101-3p to regulate the expression of C/EBPα and KLF6 to mediate *Mφs* polarization, promote the expression of lncRNA *Xist* in M1 *Mφs* and inhibit the expression of miR-101-3p in M2 *Mφs*, thus inhibiting the progression of breast cancer. TAMs reprogram their specific functional phenotype in the tumor environment to promote cancer progression and metastasis. Zhou et al. (16) showed that lncRNA *p21* was significantly upregulated in *Mφs*. Knockout of *P21* led to mouse double minute 2 (MDM2)-induced proteasome-dependent degradation of p53 and activated the NF-κB/STAT3 pathway, which promoted *Mφs* polarization to pro-inflammatory M1 and accelerated the progression of breast cancer. These results show that lncRNA *p21* plays a key role in regulating the function of TAMs and can be a new therapeutic target for breast cancer.

In addition, after Zong (19) successfully induced M2 polarization by IL-4/IL-13 experimentally, the RNA level of lncRNA *SNHG1* determined using by qRT-PCR was increased, indicating that this lncRNA is involved in M2 polarization. Further experiments showed that lncRNA *SNHG1* knockout inhibited phosphorylation of STAT6 and indirectly weakened M2 polarization. Subsequent transplantation of *Mφs* mixed with other cells accelerated the growth of breast cancer cells. The lncRNA *SNHG1* regulates M2 polarization to affects cell growth and invasion. LncRNA *00337* (20) and lncRNA *00514* (21) have similar mechanisms and can both affect the development of breast cancer by regulating M2 TAMs polarization.

### Other Systemic Cancers

In addition to the above common cancers, *Mφs* polarization is involved in the pathological mechanisms of osteosarcoma and brain tumors. Osteosarcoma is common in children and adolescents. The lncRNA *LOC100129620* can promote the proliferation and migration of osteosarcoma cells *in vivo* (33) by binding to miR-335-3p and regulating cyclin-dependent kinases 6 (CDK6), thus promoting angiogenesis and *Mφs* polarization. In contrast, *LOC100129620* gene knockout decreased the infiltration and M2 TAMs polarization, and inhibited the development of osteosarcoma. In glioblastoma multiforme, Zhang found (38) that coagulation factor X (FX) was highly expressed and positively correlated with the concentration of TAMs. FX is secreted in the tumor microenvironment, has a strong chemotactic effect on *Mφs*, promotes the phosphorylation and activation of ERK1/2 and AKT in *Mφs*, and stimulates *Mφs* to M2 polarization. In addition, FX was identified as the target gene of miR-338-3p. The lncRNA *CASC2c* interacts with miR-338-3p and binds with FX to jointly inhibit the expression of FX and finally inhibit M2 polarization.

In summary, lncRNAs and *Mφs* play important role in multi-system cancers such as the respiratory, digestive, and reproductive systems. Through the analysis of the results of the above studies, it can be found that the mechanism of lncRNAs regulating *Mφs* polarization is mainly through the lncRNA-miRNA-mRNA axis. A large number of animal experiments and clinical trials have confirmed that M2 TAMs play an important role in tumor growth, invasion and metastasis, while M1 TAMs can inhibit tumor formation. It is also found that M2 polarization occurs more frequently and the mechanism of action is more complex than that of M1 in the occurrence and development of cancers. The presence of a large number of M2 *Mφs* in tumor tissue indicates a poor prognosis, which has a positive reference value for the clinical treatment of M2 *Mφs*. In the TME, TAMs produce a polarization response, secrete inflammatory factors and chemokines, regulate immune responses, and participate in the occurrence and development of cancers, which is important in cancer pathogenesis. Therefore, blocking *Mφs* from reaching tumor tissue, clearing *Mφs* and inhibiting the activation or repolarization of TAMs in TME has become a hot issue in the field of tumor immune research. Based on the understanding of the function of TAMs and the relationship between M1 and M2 *Mφs*, reversing the polarization of M1 to M2 *Mφs* is likely to be a key target for future tumor therapy. However, tumor treatment is a systematic project, and the effect of any single treatment is very limited. Regulating *Mφs* polarization through lncRNAs acting on different targets and pathways can provide new hope for the treatment of many types of cancers. Through the study of these lncRNAs *in vivo* and *in vitro*, TAMs can be polarized into pro-inflammatory or anti-inflammatory state, so as to develop new strategies to change the phenotype of *Mφs*. These cell therapies can be further combined with currently available chemotherapy and radiotherapy options to improve therapeutic effectiveness (113).

## LncRNAs Regulate Mφs Polarization in Inflammatory Diseases

In addition to resisting pathogens, *Mφs* play a key role in eliminating inflammation and repairing damaged tissues. The polarization response of *Mφs* is essential for immunity and dynamic balance. In the early stage of tissue injury, *Mφs* are activated to produce a pro-inflammatory effect, thereby removing cell fragments and pathogens (114). During this phase of transformation, *Mφs* change their phenotype and play a key role in inflammation regression and tissue repair (115).

Osteoarthritis (OA) is a joint degenerative disease that often occurs in the elderly, and affects joint activity and function. Currently, there is no efficient and standardized treatment. A large number of studies has shown that many inflammatory cells and inflammatory factors, such as IL-6, IL10, TNF-α, are involved in the pathogenesis of OA (116). For example, M1 *Mφs* secrete pro-inflammatory cytokines. Under the influence of the inflammatory microenvironment, M1 *Mφs* are activated and secrete high levels of inflammatory mediators, infiltrating synovial tissues and chondrocytes, leading to apoptosis and accelerating the development of OA, which is considered as a key factors regulating the pathogenesis of OA (117). The results of a previous study suggested (58) that osteopontin (OPN) affect M1 polarization. Overexpression of OPN can promote M1 *Mφs* polarization, produce pro-inflammatory cytokines and degrading enzymes, and act on chondrocytes to exert a cytotoxic effect. Further experiments showed that lncRNA *XIST* acts on OPN indirectly. It binds to miR-376c-5p competitively through the competing endogenous RNA mechanism, thus weakening the inhibition of miR-376c-5p on OPN, and then affects M1 polarization. In addition, Xist gene knockout affects M1 *Mφs* and chondrocyte apoptosis. The effect of the lncRNA *XIST*/miR-376c-5p/OPN axis on M1 *Mφs* and the inflammatory microenvironment may be a new mechanism of apoptosis in OA chondrocytes. A similar mechanism was found in the lncRNA *IGHCγ1* (90). This lncRNA is mainly located in the cytoplasm of *Mφs* and competitively binds to miR-6891-3p *via* the NF-κB signaling pathway, and regulates TLR4 to inhibit *Mφs* proliferation and inflammation, ultimately affecting OA development.

M1 and M2 polarization play important roles in lung inflammation and injury (118). Targeting *Mφs* polarization and affecting the inflammatory microenvironment may be useful for treating pneumonia. LncRNA *GAS5* plays a key role in cell proliferation, invasion, apoptosis, and *Mφs* polarization (119). In a study of childhood pneumonia, Chi et al. (53), showed that overexpression of lncRNA *GAS5* inhibits JAK2/STAT3 signal transduction, which promotes M1 polarization. The specific mechanism involves the miRNA-mRNA action axis: GAS5 combined with miR-455-5p can promote the expression of SOCS3, and then inhibit the JAK2/STAT3 pathway, thus promoting phenotypic polarization of *Mφs* from M2 to M1. These findings have potential for the development of therapeutic strategies against pneumonia. The mechanisms by which lncRNAs regulate M1 or M2 polarization in the pneumonia microenvironment are similar; for example, lncRNA *p21* and lncRNA *NKILA* promote M2 polarization through NF-κB signaling pathway (54, 64). Moreover, through the PI3K/AKT pathway, lncRNA *Gm16410* reduces the expression of TNF-α in *Mφs*, inhibits inflammation, and stimulates polarization of *Mφs* induced by PM2.5, which participates in the activation of *Mφs* and process of inflammation (78). In addition, these effects have been described in numerous studies.

*Mφs* have also been widely reported to be involved in the pathogenesis of cardiovascular inflammatory diseases. Viral myocarditis is a type of heart inflammatory disease caused by viruses, that can lead to heart failure in severe cases. There is increasing evidence that *Mφs* polarization plays an important role in coxsackievirus B3-induced viral myocarditis. Zhang et al. (67) found that lncRNA *AK085865* can increase the susceptibility to viral myocarditis by regulating the *Mφs* polarization. It specifically binds to interleukin enhancer-binding factor (ILF2) and promotes M2 *Mφ* polarization by negatively regulating the ILF2-ILF3 complex-mediated miRNA-192 pathway. Overexpression of miRNA-192 can also effectively stimulate the transformation of myocardial infiltrating *Mφs* to the M2 phenotype. This reveals the key mechanism by which lncRNA *AK085865* regulates *Mφs* polarization and act on myocarditis. LncRNA *MEG3* participates in the regulation of M1/M2 *Mφs via* another pathway (68); it regulates tumor necrosis factor receptor-associated factor 6 (TRAF6) expression by binding to miR-233. Inhibitory *MEG3* can downregulate the NF-κB pathway; inhibited the expression of iNOS, TNF-α, and CD86; reduced the expression of M1 *Mφs*; promoted the expression of Arg-1 and Fizz-1; increased the M2 polarization, and finally reduced cardiomyocyte injury.

In traumatic diseases, lncRNA *GBP9* regulates *Mφs* polarization and reduces the inflammatory response after spinal cord injury through corresponding pathways. Studies have shown that after spinal cord injury, *Mφs* first differentiate into M1 *Mφs* under the stimulation of lipopolysaccharide and TNF-α, and TLRs on the cell surface are activated, which induces the recruitment of downstream protein myeloid differentiation factor 88, activates NF-κB, JAK-STAT, and other pathways; and promotes M1 to release TNF-α, CCL8, and other molecules (57, 120).

Moreover, M2 *Mφs* surface receptors that bind to IL-4 and IL-13 can promote the phosphorylation of STAT6 and stimulate M2 *Mφs*. M2 *Mφs* can highly express IL-10, transforming growth factor (TGF)-β and neurotrophic factors, which can inhibit the nerve cell apoptosis and pro-inflammatory effects of M1, as well as promote nerve tissue repair (121). Zhou et al. found (57) that lncRNA *GBP9* competitively binds to miR-34a to eliminate the inhibition of suppressor of cytokine signaling-3 (SOCS3), thus regulating STAT1/STAT6 signaling and *Mφs* polarization. After M1 polarization, M1 showed stronger phagocytosis and antigen presentation ability. In M1 *Mφs*, lncRNA *GBP9* overexpression significantly decreased the expression of p-STAT1, SOCS3, IL-6, and IL-12, whereas in M2 *Mφs*, the same overexpression increased the concentration of SOCS3 and decreased the production of p-STAT6, IL-10, and TGF-β1, indicating that lncRNA *GBP9* overexpression promoted M1 polarization. In contrast, after silencing lncRNA *GBP9*, the M2 polarization response was enhanced, nerve repair factor was secreted, and spinal cord injury was improved.

## LncRNAs Regulate Mφs Polarization in Infections

M1 *Mφs* have a strong cytotoxic effect on infected cells and are resistant to infection. Human immunodeficiency virus (HIV) infection poses a challenge to human health. Studies have shown that *Mφs* polarization is associated with HIV infection and that these cells are immune to virus-induced cell death. M1 *Mφs* play an important role in early antiviral immune responses and late restorative responses in the process of HIV infection (122). These *Mφs* show good prospects for treating HIV infection by blocking the aggregation of *Mφs* at the inflammatory site or changing the *Mφs* polarization. Boliar et al.’ data (92) showed that the expression of lncRNA *SAF* was significantly upregulated in HIV-1-infected *Mφs*. Downregulation of *SAF* can increase the activity of caspase-3/7 in virus-infected *Mφs*. This caspase-induced apoptosis occurs only in HIV-1-infected *Mφs*, but not in other cells, resulting in a significant reduction of HIV-1 virus in *Mφs*. Therefore, targeting of lncRNA *SAF* is a potential method for inducing the death of *Mφs* infected by HIV-1.

## LncRNAs Regulate Mφs Polarization in Metabolic Diseases

Recent studies revealed that the mechanism by which lncRNAs regulate *Mφs* are important in the development of metabolic diseases, such as diabetes. Diabetes mellitus is related to insulin resistance. It can promote inflammatory responses and induce neurovascular complications through the proliferation, polarization, and dysfunction of *Mφs* (123). Das et al. (86) studied the role of lncRNA *Dnm3os* in diabetes. The results showed that in *Mφs*, a decrease in nucleolin and overexpression of *Dnm3os* changed the modification of intracellular histone, enhanced the promoter H3K9ac, recruited histone acetyltransferase, and targeted upregulated the genes related to inflammation and immune response such as NKx3-2 AP1, STAT, and IRF1, which promoted phagocytosis, whereas knockout of *Dnm3os* weakened these responses. In addition, nucleolin gene knockout increased the expression of pro-inflammatory factor IL-6 in *Mφs*, which is further enhanced by lncRNA *Dnm3os*-regulated *Mφs*. Diabetic peripheral neuropathy (DPN) is the main complication of type 2 diabetes mellitus. It has been reported that the expression of M1 *Mφs* in patients with DPN is significantly increased and mediates the secretion of inflammatory factors such as IL-1 and IL-6, which promotes the occurrence and development of DPN (4). Further studies (52) showed that in DPN, lncRNA *HCG18* competitively binds to miR-146a and regulates TNF receptor associated factor 6 (TRAF6) to promote M1 polarization and inflammatory factor secretion. MiR-146a can negatively regulate the expression of TRAF6, whereas inhibition of TRAF6 can reverse the promoting effect of *HCG18* on M1 *Mφs*. *HCG18* stimulates *Mφ* activation *via* the TRAF6/NF-κB pathway. Therefore, lncRNA *HCG18* can promote the proliferation of M1 *Mφs* in DPN and accelerate the development of disease by regulating the miR-146a/TRAF6 axis.

## LncRNAs Regulate Mφs Polarization in Autoimmune Diseases

Rheumatoid arthritis (RA) is a common autoimmune disease with a complex pathogenesis, and its incidence is increasing. In RA, TNF-α produced by M1 *Mφs* can trigger synovial cells to produce cytokines, leading to the development of arthritis. Infiltrating *Mφs* in the joint synovium plays an important role in the pathogenesis of RA, and the degree of infiltration is related to the activity of the disease and degree of joint erosion (124). A study showed (61) that silencing of lncRNA *H19* altered the expression of lipopolysaccharide in *Mφs* from patients with RA, induced M1 polarization, and reduced the expression of factors such as IL-6 and CXCL10. However, overexpression of *H19* showed the opposite effects by promoting M1 polarization and aggravating arthritis in mice. In practice, lncRNA *H19* is highly expressed in patients with RA. Increased expression of KDM6A, promotes M1 polarization and aggravates arthritis. Multiple sclerosis (MS) is a chronic inflammatory disease of the central nervous system (CNS) characterized by extensive inflammation, demyelination and axonal loss (125, 126). Lesions often occur in the brain and spinal cord. Due to unknown etiology and complex pathophysiological mechanisms, the etiology and pathogenesis of MS have not been fully elucidated (127, 128). Microglia can be divided into M1 and M2 functional subtypes; they play a central role in inflammatory process and neuronal destruction in MS (129). It has been found that M2 polarized microglia can promote neuronal survival, neurite growth and oligodendrocyte progenitor cell (OPC) differentiation (130, 131). In epigenetics, lncRNA *Gas5* is an epigenetic regulator of microglial polarization. It is highly expressed in microglia of patients with MS and can inhibit M2 polarization of microglia. The specific mechanism is that *Gas5* suppresses the transcription of TRF4, a key factor regulating M2 polarization, through recruiting the polycomb repressive complex 2 (PRC2), thus inhibiting M2 polarization. Therefore, this study suggests that lncRNA *Gas5* may be a promising target for the treatment of MS (76).

## Conclusion

*Mφs* constantly convert between two extreme polarization states, M1 and M2 *Mφs*. *Mφs* are involved in the pathogenesis and progression of many diseases. Identifying the key regulators of *Mφs* polarization is important for developing treatments for diseases. An increasing number of lncRNAs has been identified in the process of *Mφ* polarization. There is a close relationship between lncRNAs and *Mφs*, which play an important regulatory role in *Mφs*. Their multifunctional capabilities and heterogeneous working mechanisms make them potential targets for treating diseases. In conclusion, lncRNAs exhibit tremendous effects on *Mφs* differentiation and polarization. The interaction between lncRNAs and *Mφs* is involved in the development of multiple systemic diseases and provides useful targets for the interference of macrophage involved disorders.

However, compared with the study of small molecule RNA, lncRNAs are still in the initial stage, and their function and regulation mechanism still need to be further clarified. In order to fully realize the therapeutic value of lncRNAs and the development of corresponding therapeutic drugs, there are still some difficulties to be overcome. First of all, we need to improve the method of transporting lncRNAs to specific areas. Because of the long length of lncRNA, it is difficult to deliver full-length lncRNAs, so we need an accurate and efficient means of transport to ensure the stability of lncRNAs. Secondly, we need to establish a complete, detailed and specific lncRNAs database to record the specific regulatory cell types and target genes of each lncRNAs. Finally, we need to solve the difficulty of finding homologous lncRNA. Especially in animal experiments, due to the epigenetic differences between animals and humans, the human lncRNAs may not exist in animals. This requires techniques such as gene knockout to build animal models, which makes the experiment more difficult. In recent years, with the deepening of research, the experimental techniques related to lncRNAs have been continuously developed, an increasing number of lncRNAs have been found, and play an important role in the occurrence and development of various diseases, and can be used as genes related to diagnosis and treatment. In particular, there is an increasing number of studies on lncRNAs regulating *Mφs* polarization and participating in the development of various diseases, which provides a new research content for future research. On the basis of fully understanding the mechanism of action, appropriate genetic engineering drugs are developed to achieve the purpose of prevention, diagnosis and treatment.

## Author Contributions

PJ wrote the manuscript. XL conceived the study. All authors have reviewed and approved the final version of the manuscript.

## Conflict of Interest

The authors declare that the research was conducted in the absence of any commercial or financial relationships that could be construed as a potential conflict of interest.

## Publisher’s Note

All claims expressed in this article are solely those of the authors and do not necessarily represent those of their affiliated organizations, or those of the publisher, the editors and the reviewers. Any product that may be evaluated in this article, or claim that may be made by its manufacturer, is not guaranteed or endorsed by the publisher.
